# Chloroform

**Published:** 1857-09

**Authors:** J. S. Weatherly

**Affiliations:** Montgomery, Alabama


### ARTICLE IV.

*Chloroform* By J. S. WEATHERLY, M. D., of Montgomery, Ala.

No remedy in the Materia Medica, I opine, is more potent for good, if properly used, than Chloroform; and yet, it seems to be distrusted, and to be entirely discarded by a large number of the profession. They say that its use is fraught with danger. So it is, and so is the indiscriminate use of any powerful remedy. Suppose the ghosts of all those dead from the abuse of opium, mercury, &c., were arrayed in solemn order, and made to pass before the vision of our profession! Methinks the line of Banquo's ghosts would be small in comparison to the long line that would shake their bony fingers in solemn warning to the young Galens of our profession; to beware of these potent remedies. And yet, I presume it would take more than the eloquence of a Weller, sr., to his son Samivel, "to beware of the vidders," added to the testimony of ghosts, to induce us to lay aside these powerful and excellent remedies. Who would be willing to see his patient writhing in pain from spasm of the colon, and not give him opium, for the reason that it is a powerful remedy and must not be tampered with? We want powerful remedies to fight disease with. It would be foolish to try to extinguish a fire by blowing at it with our breath, when we know that water is its natural antagonistic force, and is sure to subdue it if used in sufficient quantities. Yet, some will look on a poor woman going into convulsions, from an irritable os uteri, and never use the only remedy that offers the greatest certainty of relief.

Let us digress a little, and consider for a few moments the terror of the lying in chamber. A delicate, nervous woman, after suffering a continually exciting, or more properly, an exhausting influence, upon her nervous system for nine months, arrives at the proper period for her confinement. The os uteri commences to dilate, pain comes on, every nerve is quivering with excitement. The influence is carried to the brain and spinal marrow. The machine is already out of order from over-work. The natural smooth flowing nervous current is disturbed, and the woman is thrown into convulsions. Just as we have convulsions from nervous exhaustion after bleeding, or from any other cause which detracts the natural stimulus from the brain. The physician is sent for; it is a case of puerperal convulsions. His mind reverts back to the ponderous tomes in his library, the great authors from whom he has been drinking in medical lore, and who from their misty heights have been shining upon and illuminating his pathway of darkness. He is lost in thought for a moment, and a moment only. Perhaps he is thinking of the awful responsibility of his position. Life! human life! is depending on him; he is thus to battle with death itself. Well may he think. Ah! he has it now; the *books* say bleed for convulsions, and he bleeds. For what? Because the books say so, and he wants his patient to die according to authority. What is the consequence? The bleeding exhausts still more the brain and nervous system, renders them still more susceptible, and the convulsions increase. Another bleeding, and more convulsions. The woman dies, from what? The convulsions or the bleeding. Suppose now (as I have had occasion to do in some five or six cases,) a handkerchief moistened with chloroform is applied to the nose; the woman breathes; those irritable, worn-out nerves quiet down; she sleeps gently and quietly—"sleep that sometimes shuts up sorrow's eyes"—brings rest to the nervous system, and "affords the only boon the wretched mind can feel." The influence of the chloroform is carried to every part of the system; the rigid os uteri dilates, and by a little careful manipulation the patient is delivered of a plump crying babe. All is well now; the patient and friends gratified, and the physician feeling as if he had been the means of saving life. Again: I have seen patients (lying in) who were worn out by irregular and spasmodic pains, where no progress was being made towards delivery, and the patient gradually sinking from nervous exhaustion. They were placed under the influence of chloroform, and as if by magic, the pains would become expulsive, and the woman be delivered quickly and safely. This is no fancy sketch. I am satisfied from experience that we have no remedy so sure of controlling convulsive diseases, especially in females and children, as chloroform. I, of course, do not advocate its indiscriminate use. There are cases where it would not be proper to use it, and others where it comes in merely as an adjunct. If convulsions are dependent on inflammation, of course use remedies for subduing it; but in nine cases out of ten, especially in puerperal convulsions, there is anything else except inflammation. The nervous system is exhausted from continued excitement. You wish to rest it; for you know by that means it will be invigorated and resume its natural actions. And among all the remedies for procuring this rest, quickly and speedily, none are equal to chloroform. I intended to enumerate other forms of disease, for which I have used chloroform, but have not the time at present.

